# Lower HDL-C levels are associated with higher expressions of CD16 on monocyte subsets in coronary atherosclerosis

**DOI:** 10.7150/ijms.47998

**Published:** 2020-08-01

**Authors:** Yang Xiang, Bin Liang, Xiaokang Zhang, Fang Zheng

**Affiliations:** Center for Gene Diagnosis, and Clinical Lab, Zhongnan Hospital of Wuhan University, Donghu Road 169, Wuhan 430071, China.

**Keywords:** coronary atherosclerosis, HDL-C, CD14, CD16, monocyte subsets

## Abstract

**Background:** Increased expressions of CD16 on classical monocytes precede their transition to intermediate monocytes. Thus far, the influence of lipids on the expression of CD14 and CD16 on monocyte subsets in coronary atherosclerosis (CA) remains unclear. The aim of this study was to investigate the underlying association between blood lipids and the expression of CD14 and CD16 on monocyte subsets.

**Methods:** This study enrolled 112 healthy controls and 110 CA patients. Monocyte subsets [CD14^++^CD16^-^ (classical), CD14^++^CD16^+^ (intermediate) and CD14^+^CD16^++^ (non-classical)] were analyzed by flow cytometry. Median fluorescent intensity (MFI) was used to evaluate the expression levels of CD14 and CD16 on monocyte subsets.

**Results:** Compared with the control group, the expression of CD16 was significantly increased on all three monocyte subsets in the patient group. Correlation analysis revealed that serum HDL-C was inversely associated with the expression of CD16 on intermediate monocytes after Bonferroni correction in the control group. In addition, a significant decrease in classical monocytes and an increase in intermediate monocytes were detected in patients. In linear regression analysis, intermediate monocytes showed an inverse association with serum HDL-C in the control group. Although CD14 was correlated with serum TC and HDL-C, there was no statistical difference in CD14 expression between the two groups.

**Conclusion:** Low serum HDL-C may induce upregulation of CD16 on classical monocytes, which may in turn lead to the increase of intermediate monocytes in coronary atherosclerosis patients.

## Introduction

Atherosclerosis is a chronic vascular inflammatory disease with complex etiology 1. It is the pathological basis of coronary atherosclerosis (CA), which remains a worldwide medical problem 2.

Monocytes play vital roles in the progression of atherosclerosis 3. In humans, based on the expression of CD14 and CD16, monocytes were normally divided into classical (CD14^++^CD16^-^), intermediate (CD14^++^CD16^+^) and non-classical (CD14^+^CD16^++^) monocytes 4. These three monocyte subsets have different functions in atherosclerosis 5. Besides, the functional phenotype of monocytes can change in response to environmental stimuli, causing significant changes in the proportion of monocyte subsets 6, 7.

Recent study revealed the alterations in the proportion of monocyte subsets in coronary artery disease 8. According to literatures, abnormally increased intermediate monocytes can independently predict cardiovascular events and are associated with the prognosis of atherosclerotic cardiovascular disease (ASCVD) 9, 10. Dyslipidemia is the first risk factor for CA patients 11. And several studies have revealed the association between lipids including TC, TG, LDL-C, small HDL and HDL-C *etc.* and the proportion of monocyte subsets 12-14.

Gating strategy based on CD14 and CD16 is the most popular classification method for the monocyte subsets among studies on coronary atherosclerosis 15. The expression levels of CD14 and CD16 on monocyte subsets have obvious individual specificity 16. And CD14 and CD16 expression levels will change with environmental stimuli 17, 18. Abnormally expressed CD14 and CD16 molecules on monocytes have been detected in coronary artery disease 19. Furthermore, alterations in the expression of CD14 and CD16 on monocyte subsets are related to the changes in the proportion of monocyte subsets. For instance, with increased expressions of CD16 and CD14, shifts of classical and non-classical monocytes to intermediate monocytes were observed in CAD patients, respectively 20.

Thus far, the effects of lipids on the expression of CD14 and CD16 on monocyte subsets remain unclear. Therefore, the aim of this study was to explore whether blood lipids are related to the expression of CD14 and CD16 on monocyte subsets, which may in turn lead to the changes in the proportion of monocyte subsets in coronary atherosclerosis patients.

## Materials and Methods

### Study population

The case-control study was approved by the Medical Ethics Committee of Zhongnan Hospital of Wuhan University (approval number 2018017, Wuhan, China). Informed consent was obtained from all participants prior to entering the study. The study was conducted in accordance with the Declaration of Helsinki.

We recruited 110 CA patients from Zhongnan Hospital of Wuhan University from July to August, 2019. All patients underwent coronary angiography and were divided into two subgroups: stenosis >50% (n = 30) and stenosis <50% (n = 80) groups. Patients with stenosis >50% were defined as coronary atherosclerotic plaque formation, with stenosis over than 50% in at least one major coronary artery; those patients with stenosis less than 50% were grouped to stenosis <50% group. At the same time, 112 healthy control subjects were randomly selected from the same hospital. Based on medical history and clinical examination, people with no history of cardiovascular or other chronic diseases were selected as healthy controls. All enrolled subjects met the following exclusion criteria: autoimmune diseases, cancer, severe infections, cerebral infarction, congenital heart disease, myocardial hypertrophy, myocardial bridge, non-diabetic endocrine diseases and other serious organic diseases. The clinical characteristics and laboratory data of patients and controls were collected. Gensini Scores was calculated based on coronary angiography and was used to evaluate the severity of coronary atherosclerosis.

### Flow cytometry analysis

The whole blood samples were collected using EDTA-anticoagulated vacutainer tubes. Blood samples were analyzed within 3 h after collection. Flow cytometry analysis was performed using the Beckman CytoFLEX flow cytometer (Beckman Coulter, CA, USA). About 100 μL whole blood was firstly added into a 2.0 ml centrifuge tube. Subsequently, the blood samples were stained with 25 μL mixture of 3 mouse anti-human monoclonal fluorochrome-conjugated antibodies [10 μL anti-CD14-PE (BD, NJ, USA), 10 μL anti-16-FITC (BD, NJ, USA), and 5 μL anti-CD86-BV421 (BD, NJ, USA)]. Tubes were gently mixed and incubated at 4 ° C in the dark for 25 minutes. Then the red blood cells were lysed using 1× BD Lysing Buffer (BD, NJ, USA) at 4 ° C in the dark for 5 minutes. After red blood cells were fully lysed, tubes were immediately centrifuged at 4 ° C, 180×g for 5 minutes. The supernatant was aspirated and discarded. About 1.0 mL of 1× PBS buffer was added into the tube to resuspend the leukocytes. Then the tubes were immediately centrifuged at 4 °C, 180×g for 5 minutes. The supernatant was aspirated and discarded to remove the residual cell debris. After that, 500 μL of 1× PBS buffer was added into the tube to resuspend the leukocytes and all the samples were analyzed within 30 min.

### Gating strategy for monocyte subsets

**Figure 1:**
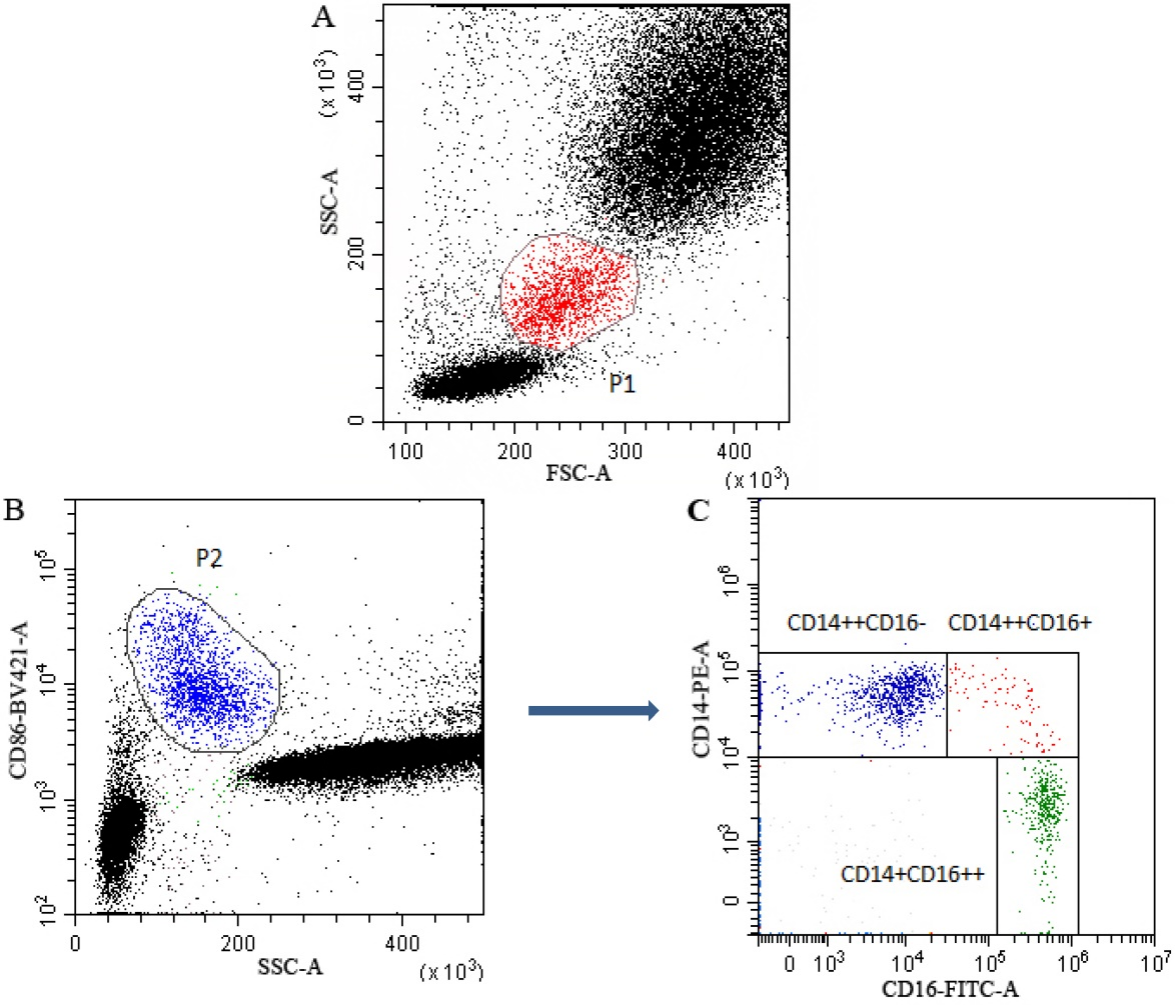
** Multiparametric flow cytometry gating strategy for monocyte subsets classification.** P1 gate represented the monocytes in leukocytes in FSC/SSC dot plot (**A**). CD86 was used to identify monocytes and the CD86^+^ monocytes (P2) were firstly gated in SSA/CD86 dot plot (**B**). Subsequently, in the CD14/CD16 dot plot, monocytes were further gated into classical (CD14^++^CD16^-^), intermediate (CD14^++^CD16^+^) and non-classical (CD14^+^CD16^++^) subsets (**C**).

Gating strategy for monocyte subsets was presented in Figure 1. Monocytes (P1) in leukocytes in FSC/SSC dot plot were presented in Figure 1A. In CD86/SSC dot plot, CD86 positive cells (P2) were firstly gated to identify monocytes (Figure 1B). Subsequently, in the CD14/CD16 dot plot, based on the expression of CD14 and CD16, the identified monocytes were divided into classical (CD14^++^CD16^-^), intermediate (CD14^++^CD16^+^) and non-classical (CD14^+^CD16^++^) subsets (Figure 1C).

The expression levels of CD14 and CD16 were quantified by flow- cytometrically as median fluorescence intensity (MFI). Fluorescence was standardized using multiple peaks rainbow calibration beads (Spherotech, Chicago, USA) to ensure the reproducibility and comparability of median fluorescence intensity (MFI) throughout the study period, as previously described 14. CytExpert (Beckman Coulter, CA, USA) software version 2.0 was used to analyze the monocyte subsets and calculate the MFI of the CD14 and C16 expression.

### Statistical analysis

Based on the distributions, continuous variables were properly presented as mean ± SD, or as median (inter-quartile range). Categorical variables were presented as frequencies (n). Comparisons between continuous variables in different groups were performed by student's* t* test or Mann-Whitney *U* test. Chi-squared test was used to compare categorical variables between the groups. *Spearman* or* Pearson* bivariate correlation analysis were used to analyze the correlations involved in this study. Linear regression analysis was used to analyze the association between monocyte subsets and lipid profiles. *Bonferroni* correction was used to adjust for multiple testing. SPSS software version 17.0 (SPSS, Chicago, USA) and GraphPad Prism 8.0 (GraphPad, San Diego, USA) were used for statistical analysis. Statistical significance was accepted at *P* < 0.05 (two-tailed).

## Results

### Baseline characteristics of the subjects

Baseline characteristics were presented in Table 1. Age and sex composition showed no significant differences between the two groups (both *P >* 0.05). Coronary atherosclerosis patients had higher proportion of diabetes (19.1%) and hypertension (48.2%) medical histories (both* P* < 0.05), and higher FPG, TG, leukocytes, neutrophils and monocytes, but lower HDL-C and lymphocytes (all *P* < 0.05).

In the subgroup analysis, both the CA subgroups differed from controls in a consistent manner to the CA group as a whole. Except for Gensini Scores (*P <* 0.0001), no significant differences were found between the two subgroups (all *P* > 0.05).

### Distributions of monocyte subsets in different groups

**Figure 2:**
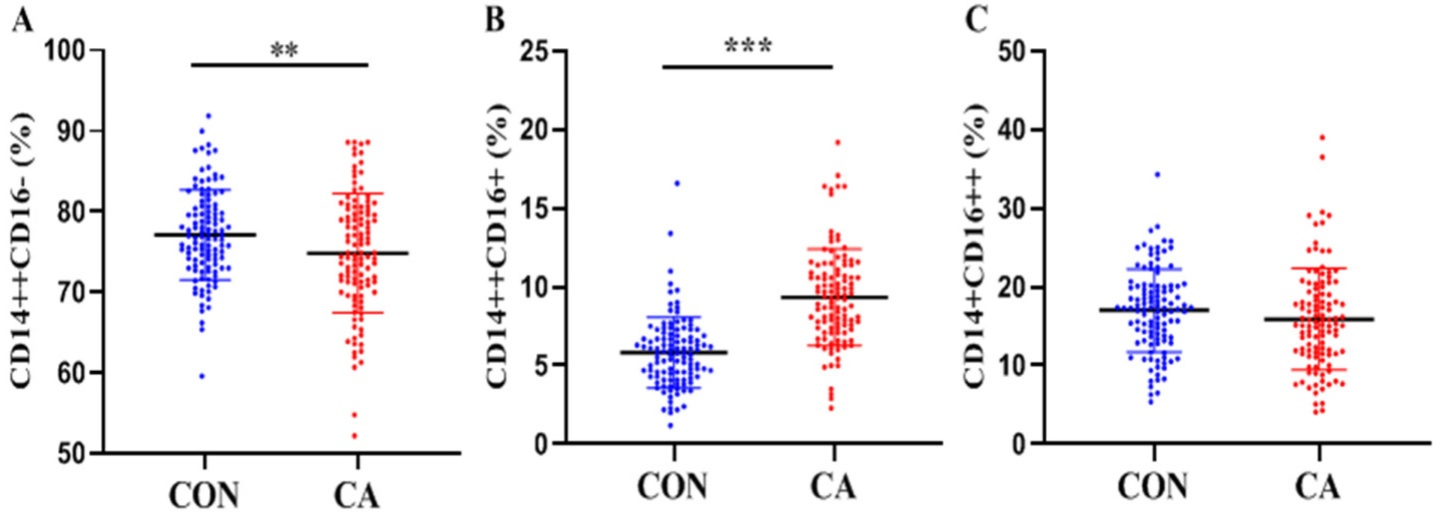
** Distributions of monocyte subsets in the control and CA groups.** Proportions (percentage) of classical (CD14^++^CD16^-^ (**A**), intermediate (CD14^++^CD16^+^ (**B**) and non-classical (CD14^+^CD16^++^ (**C**) monocytes in the control and CA groups were shown in scatter plot graphs. **P<0.01, ***P<0.001.

As shown in Table 1 and Figure 2, significantly decreased classical monocytes (*P <* 0.01, Figure 2A) and significantly increased intermediate monocytes (*P <* 0.0001, Figure 2B) were detected in patients. Non-classical monocytes showed no statistical difference between patients and controls (*P >* 0.05, Figure 2C).

In the subgroup analysis, both the CA subgroups differed from controls in a consistent manner to the CA group as a whole. In addition, the proportion of monocyte subsets in the two subgroups showed no statistical differences (all *P* > 0.05).

### The expression of CD16 was increased on all monocyte subsets in patients

Compared with the control group (Table 2), the expression of CD16 on all three monocyte subsets was significantly increased in the CA group (all *P <* 0.05). There were no significant differences in the expression of CD14 on the same monocyte subsets between CA and control groups (all *P* > 0.05). In the subgroup analysis, the expression of CD14 and CD16 showed no significant differences (all *P* > 0.05).

### CD14 expression was positively associated with HDL-C and negatively associated with TC

As shown in Table 3, in the control group, a *P* value < 0.0017 (0.05/30) was considered statistical significant (Bonferroni correction for multiple testing). HDL-C was positively correlated with CD14 expression on intermediate monocytes (r = 0.351, *P* = 0.0001). HDL-C, TC and lymphocytes were trended to correlate with CD14 expression on classical monocytes (all *P* > 0.0017).

In the CA group (Table 3), a *P* value < 0.0015 (0.05/33) was considered significant. CD14 expression on non-classical monocytes was negatively correlated with TC (r = - 0.335, *P* = 0.0003) and positively correlated with total monocyte count (r = 0.324, *P* = 0.0006).

### CD16 expression was inversely associated with HDL-C

In the control group (Table 4), a *P* value < 0.0025 (0.05/20) was considered significant. Serum HDL-C was inversely associated with CD16 expression on intermediate monocytes (r = - 0.311, *P* = 0.0008).

In the CA group (Table 4), the expression of CD16 on non-classical monocytes was trended to inversely correlate with serum HDL-C (r = - 0.237*, P* = 0.0128 > 0.0023).

### Negative correlation between intermediate monocytes and HDL-C

For this analysis, a *P* value <0.0042 (0.05/12) was considered significant (Table 5). In the control group, linear regression analysis revealed a negative correlation between intermediate monocytes and serum HDL-C (β = -0.386, *P =* 0.002). No significant correlations were found between monocyte subsets and lipid profiles in CA group (all *P* > 0.05). In the control and CA combined group, serum HDL-C showed an inverse correlation with intermediate monocytes (β = -0.465, *P <* 0.0001).

### Monocyte subsets do not associate with CA severity

**Figure 3:**
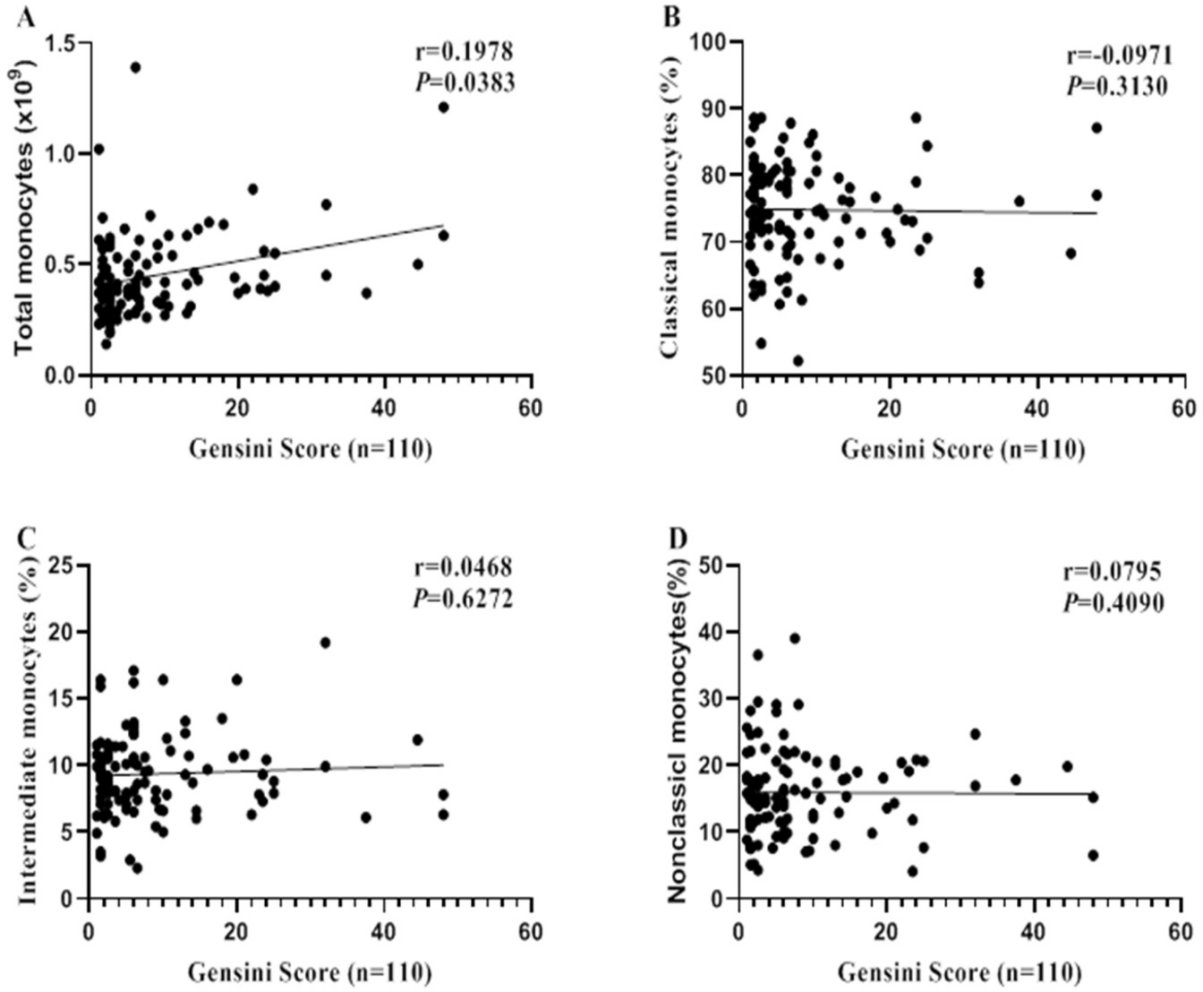
** Correlations between monocyte (subsets) and CA severity.** Spearman correlation analysis was performed to reveal the relationships between monocyte (subsets) and CA severity (Evaluated by Gensini Score).

Spearman correlation analysis was performed to reveal the relationship between monocytes and CA severity (evaluated by Gensini Scores). Total monocyte count was correlated with CA severity (r = 0.1978, *P* = 0.0383; Figure 3A). However, no significant correlations were found between monocytes subsets and CA severity (all *P* > 0.05, Figure 3B-D).

## Discussion

In the present study, the most important finding was that there was a negative correlation between HDL-C and the expression of CD16 on monocyte subsets. In addition, we detected increased intermediate monocytes in coronary atherosclerosis patients, and found an inverse association between serum HDL-C and intermediate monocytes in the control subjects.

Large epidemiological studies provided compelling evidence of a negative relationship between serum HDL-C and coronary atherosclerosis risk. Cholesterol efflux and anti-inflammatory were two important atheroprotective properties of HDL-C 21. As cholesterol efflux mediator, low serum HDL-C was demonstrated to be associated with elevated intermediate monocytes, which independently predicted the subsequent cardiovascular events in CKD patients 14. Furthermore, under dyslipidemia stimuli, intermediate monocytes exhibit more inflammatory properties than classical monocyte by higher TNF-α, IL-1β and IL-6 production, and among which IL-1β was inversely associated with serum HDL-C 22. The expansion of intermediate monocytes may reflect an inflammation state of atherosclerosis patients.

However, the underlying effects of lipids on the expression of CD14 and CD16 on monocyte subsets remain unclear. In this study, we firstly quantified CD14 and CD16 expressions and found upregulated CD16 expression on all monocyte subsets in patients. Beyond monocyte subset frequencies, increased CD16 expression may also relate to the disease risk. For instance, in T2D patients, although there was no alteration in the proportion of monocyte subsets, a higher CD16 expression was detected on all three monocyte subsets 20. Additionally, deletion of CD16 in mice can significantly inhibit the progression of atherosclerosis 23. CD16 is involved in the pathogenesis of atherosclerosis, and increased CD16 expression may provide additional information for assessing the risk of atherosclerosis.

As for the reason for the inverse association between HDL-C and CD16 expression, we speculated that the monocytopoiesis caused by lower HDL-C might be the reason. Low HDL-C induced the impairment of cholesterol efflux 14. As reported, in atherosclerotic ApoE-/- mouse, cholesterol efflux can regulate monocytosis, while infusion of reconstituted HDL treatment reduced monocytosis 24. Under the premise of detecting increased total monocyte count and decreased HDL-C in patients, we assume that HDL-C dysregulation may promote monocytosis during atherosclerosis progression. Part of newly generated monocytes may up-regulate expression of CD16 in response to HDL-C decrease, which may leads to a general increase in the expression of CD16 on all monocyte subsets.

Since monocyte differentiation trajectory revealed that classical monocytes that first originated in the bone marrow will sequentially differentiate into more matured intermediate and non-classical subsets 25. And during this process the expression of CD16 was gradually increased. The shift of classical to intermediate monocytes is accompanied by increased CD16 expression, which may be a reflection of the maturation process of classical monocytes. Low HDL-C may induce the up-regulation of CD16, which may in turn promote the maturation of monocytes. This might be responsible for the decreased classical and increased intermediate monocytes in patients in this study. However, we should note that the current classification strategy based on CD14/CD16 is classical but not sufficient to clearly distinguish monocyte subsets. Constantly discovered more markers for specificity of monocyte classification, such as CD33, CD64 and HLA-DR may help us better identify the expanded monocytes 26. In addition, the application of emerging technologies, such as single-cell sequencing, may be beneficial in elucidating the origin of expanded cells 27.

In keeping with previous studies, we confirmed that there was a negative correlation between serum HDL-C and intermediate monocytes in the controls, but not in the patient group. We thought this might be due to the drug effects. As we known, statins are common lipid-regulating drugs that affecting monocyte phenotypes in atherosclerosis. Effects of statins on monocyte subsets vary from drugs. Simvastatin and atorvastatin can reduce CD14/CD16 expression on LPS-stimulated monocytes, causing reduced intermediate monocytes in vitro 28. In addition, fluvastatin treatment in hypercholesterolemic patient leads to a decrease in CD14 expression, but an increase in more matured non-classical monocytes 29. Statins affect CD14 and CD16 expressions, causing changes in monocyte subsets. This may be the cause for the weakening of the association between HDL-C and CD16 expression and intermediate monocytes in patients.

Although CD14 was found to be positively correlated with HDL-C and negatively correlated with TC, there was no significant difference in CD14 expressions between the groups. As mentioned before, statins can affect CD14 expressions on monocytes 28, 29, which may be the cause for the above results. However, the mechanism needs to be further explored. In addition, Liu* et al.* pointed out that the lipid-induced epigenetic regulation mechanism mediated the expressions of CD14 on monocytes 17, which provides ideas for subsequent research.

At last, we performed a subgroup (stenosis >50% and stenosis <50%) analysis to figure out whether the altered monocytes can be used to predict the severity of coronary artery stenosis. However, our results indicate that the expanded intermediate monocytes in patients don't associate with coronary stenosis degrees, nor can it predict disease severity. The change in the phenotype of monocytes may be related to early vascular inflammation, but not plaque accumulation. However, it reminds us that in addition to patients with severe coronary atherosclerosis (with a stenosis exceeding 50%), patients in the early stage of atherosclerosis should also be concerned. Reversing the monocyte phenotype through lipid-regulating at an early stage may help prevent atherosclerosis.

Taken together, our study put forward a new insight that beyond monocyte subsets, increased CD16 expression may also provide additional information for assessing the risk of atherosclerosis. Restoring HDL-C functionality might be a way to modulating CD14 and CD16 expression, which may be a potential mechanism to reverse the abnormal monocyte phenotype and prevent coronary atherosclerosis.

There are several limitations in the present study. Firstly, the current classification strategy is relatively limited. The introduction of more markers, such as CCR2, Slan and HLA-DR, is beneficial for better differentiation of monocyte subsets. Secondly, this is a cross-sectional study that we cannot explain the regulation mechanism of lipids on the expressions of CD14 and CD16. Lastly, several clinical characteristics, such as medication information, smoking habits and body mass index were failed to collect, which was one of the limitations.

In summary, low serum HDL-C may induce upregulation of CD16 on monocyte subsets, which may in turn lead to the increase of intermediate monocytes in coronary atherosclerosis patients.

## Figures and Tables

**Table 1:** Baseline characteristics and monocyte subsets in different groups

Variables	CON (n=112)	CA (n=110)	CA patients (n=110)	
			CA (<50%, n=80)	CA (>50%, n=30)
**Characteristics**				
Sex, male (%)	59 (52.7)	54 (49.1)	37 (46.3)	17 (56.7)
Age (years)	58.00 (50.00-65.00)	57.00 (52.75-65.00)	56.00 (52.25-67.00)	61.5 (52.50-67.00)
Diabetes, yes (%)	5 (4.5)	21 (19.1)*	15 (18.8)^+^	6 (20.0)^+^
Hypertension, yes (%)	29 (25.9)	53 (48.2)*	35 (43.8)^+^	18 (60)^+^
Gensini Score	-	5.00 (2.00-10.50)	2.5 (1.5-6.0)	19.75 (12.5-25.0)^#^
FPG (mmol/L)	5.41 (5.04-5.67)	5.68 (5.20-6.52)*	5.72 (5.23-6.48)	5.61 (5.13-5.61)
TC(mmol/L)	4.64 (4.13-4.95)	4.58 (3.66-5.50)	4.65 (3.72-5.53)	4.17 (3.48-5.45)
TG (mmol/L)	1.09 (0.88-1.36)	1.30 (0.97-1.81)*	1.26 (0.95-1.77)^+^	1.49 (1.02-2.05)^+^
LDL-C (mmol/L)	2.68 (2.26-3.10)	2.71 (2.09-3.39)	2.73 (2.14-3.49)	2.59 (2.03-3.33)
HDL-C (mmol/L)	1.40 (1.26-1.66)	1.05 (0.95-1.31)*	1.05 (0.98-1.32)^+^	1.03 (0.88-1.30)^+^
Leukocytes (×10^9^)	5.43 (4.80-6.31)	5.83 (4.83-6.58)*	5.72 (4.72-6.62)	6.07 (3.51-6.56)
Neutrophil (×10^9^)	2.95 (2.56-3.54)	3.58 (2.69-4.39)*	3.58 (2.59-4.35)^+^	3.60 (2.87-4.70)^+^
Monocyte (×10^9^)	0.38 (0.31-0.46)	0.40 (0.34-0.54)*	0.40 (0.32-0.53)	0.44 (0.37-0.58)
Lymphocyte (×10^9^)	1.92 (1.65-2.29)	1.51 (1.25-1.79)*	1.51 (1.24-1.76)^ +^	1.51 (1.28-1.92)^+^
**Monocyte Subsets**				
Classical monocytes (%)	77.12±5.56	74.83±7.39*	74.65±7.77^+^	75.33±6.35^+^
Intermediate monocytes (%)	5.80 (4.30-6.90)	9.25 (7.38-10.95)*	9.10 (7.40-11.28)^+^	9.30 (6.68-10.88)^+^
Non-classical Monocyte (%)	17.30 (13.45-20.10)	15.25 (11.50-19.50)	15.15 (11.5-20.23)	16.05 (12.03-19.28)

Data are presented as mean ± SD or median (interquartile ranges) or number (%) as appropriate. *: *P*<0.05, comparison between the whole CA group and the control group; +:* P*<0.05, comparison between each subgroup and the control group; #: *P*<0.05, comparison between the two subgroups. Abbreviation: CA: coronary atherosclerosis; FPG: fasting plasma glucose; TC: total cholesterol; TG: triglycerides; LDL-C: low-density lipoprotein cholesterol; HDL-C: high-density lipoprotein cholesterol.

**Table 2:** Expression levels of CD14 and CD16 on monocyte subsets in different groups

Markers	Subsets	CON (n=112)	CA (n=110)	CA (<50%) (n=80)	CA (>50%) (n=30)
CD14	Classical	28713±11030	27637±7269	27397±7260	28276±7379
(MFI, A.U.)	Intermediate	26884±15060	26445±8411	26638±8460	25928±8401
	Non-classical	3616±1274	3659±1089	3583±1132	3864±952.3
CD16	Classical	9194 (4112-13678)	12057 (5241-16396)*	12290 (5402-16050)	11225 (3553-17112)
(MFI, A.U.)	Intermediate	129814 (100724-190326)	163529 (117865-207697)*	161268 (115393-205854)^+^	170797 (120960-213660)
	Non-classical	223682 (170117-297508)	253174 (210823-329176)*	258533 (197807-339642)^+^	247669 (225564-319953)^+^

Data are presented as mean ± SD or median (interquartile ranges) as appropriate. *: *P* < 0.05, comparison between the whole CA group and the control group; +: *P* < 0.05, comparison between each subgroup and the control group. Abbreviation: MFI: Median Fluorescence Intensity; A.U.: Arbitrary Unit.

**Table 3:** Spearman correlation between clinical characteristics and the expression of CD14 on monocyte subsets

Characteristics	CON-CD14 (n=112)						CA-CD14 (n=110)					
	Classical		Intermediate		Non-classical		Classical		Intermediate		Non-classical	
	*r*	*P*	*r*	*P*	*r*	*P*	*r*	*P*	*r*	*P*	*r*	*P*
Age (years)	0.042	0.6591	0.078	0.4112	-0.165	0.0818	0.170	0.0767	-0.022	0.8226	0.145	0.1317
Gensini Score	-	-	-	-	-	-	-0.052	0.5930	-0.036	0.7051	0.099	0.3041
FPG (mmol/L)	0.028	0.7733	0.145	0.1260	0.105	0.2707	0.109	0.2568	0.032	0.7409	0.054	0.5731
TC(mmol/L)	-0.196	0.0379^a^	-0.092	0.3331	-0.034	0.7236	-0.225	0.0182^b^	-0.012	0.9005	-0.335	0.0003^b^
TG (mmol/L)	-0.173	0.0682	-0.185	0.0511	-0.038	0.6929	-0.157	0.1007	0.008	0.9356	-0.137	0.1529
LDL-C (mmol/L)	-0.171	0.0715	-0.175	0.0643	0.091	0.3379	-0.123	0.2024	0.056	0.5626	-0.231	0.0153^b^
HDL-C (mmol/L)	0.292	0.0018^a^	0.351	0.0001^a^	0.026	0.7874	0.038	0.6940	0.016	0.8651	0.001	0.9963
Leukocytes (×10^9^)	-0.108	0.2592	0.009	0.9220	-0.038	0.6886	0.028	0.7718	0.006	0.9467	0.119	0.2167
Neutrophil (×10^9^)	0.033	0.7274	0.130	0.1723	-0.042	0.6621	0.035	0.7196	0.049	0.6127	0.133	0.1666
Monocyte (×10^9^)	-0.071	0.4566	-0.025	0.7969	0.006	0.9522	0.160	0.0942	0.027	0.7800	0.324	0.0006^b^
Lymphocyte (×10^9^)	-0.229	0.0154^a^	-0.151	0.1128	0.013	0.8906	-0.058	0.5471	-0.103	0.2841	-0.088	0.3608

After Bonferroni correction: a: in the control group, *P* < 0.0017 (0.05/30) was considered significant; b: in the CA group, *P* < 0.0.0015 (0.05/33) was considered significant. After Bonferroni correction, results still statistically significant were in bold.

**Table 4:** Spearman correlation between clinical characteristics and the expression of CD16 on monocyte subsets

Characteristics	CON-CD16 (n=112)				CA-CD16 (n=110)			
	Intermediate		Non-classical		Intermediate		Non-classical	
	*r*	*P*	*r*	*P*	*r*	*P*	*r*	*P*
Age (years)	0.080	0.4019	0.133	0.1619	-0.019	0.8405	-0.047	0.6295
Gensini Score	-	-	-	-	-0.141	0.1431	0.009	0.9272
FPG (mmol/L)	0.085	0.3742	-0.079	0.4077	-0.166	0.0827	0.013	0.8953
TC (mmol/L)	-0.156	0.0995	0.012	0.9036	0.037	0.7018	0.059	0.5400
TG (mmol/L)	0.182	0.0549	0.219	0.0203^a^	0.052	0.5901	0.059	0.5397
LDL-C (mmol/L)	0.089	0.3532	0.047	0.6251	0.001	0.9964	-0.032	0.7411
HDL-C (mmol/L)	-0.311	0.0008^a^	-0.147	0.1219	-0.086	0.3713	-0.237	0.0128^b^
Leukocytes (×10^9^)	0.051	0.5954	0.083	0.3842	-0.067	0.4899	-0.112	0.2457
Neutrophil (×10^9^)	0.021	0.8300	0.124	0.1918	-0.042	0.6618	-0.106	0.2726
Monocyte (×10^9^)	-0.088	0.3555	0.024	0.8007	0.012	0.9011	0.049	0.6095
Lymphocyte (×10^9^)	0.081	0.3967	-0.031	0.7445	0.023	0.8106	-0.104	0.2799

After Bonferroni correction: a: in the control group, *P* < 0.0025 (0.05/20) was considered significant; b: in the CA group, *P* < 0.0023 (0.05/22) was considered significant. After Bonferroni correction, results still statistically significant were in bold.

**Table 5:** Linear regression analysis between monocyte subsets and lipid parameters

Groups	Monocyte Subsets	TC		TG		LDL-C		HDL-C	
		*β*	*P*	*β*	*P*	*β*	*P*	*β*	*P*
Control (n = 112)	Classical (%)	-0.149	0.293	0.017	0.879	0.145	0.289	0.161	0.205
	Intermediate (%)	0.195	0.150	-0.182	0.087	0.014	0.916	-0.386	0.002^a^
	Non-classical (%)	0.081	0.567	0.083	0.457	-0.160	0.245	0.017	0.895
CA (n = 110)	Classical (%)	-0.171	0.410	0.125	0.302	0.020	0.912	0.101	0.373
	Intermediate (%)	0.024	0.905	-0.185	0.120	-0.052	0.774	-0.164	0.142
	Nonclassical (%)	0.165	0.427	-0.051	0.675	-0.007	0.971	-0.029	0.798
Combined (n = 222)	Classical (%)	-0.200	0.108	0.083	0.312	0.085	0.447	0.192	0.018^a^
	Intermediate (%)	0.182	0.115	-0.129	0.092	-0.096	0.350	-0.465	<0.0001^a^
	Non-classical (%)	0.124	0.324	-0.019	0.818	-0.055	0.626	0.039	0.629

After Bonferroni correction: a: *P* < 0.0042 (0.05/12) was considered significant; After Bonferroni correction results still statistically significant were in bold.
